# Constitutive activation of the EGFR–STAT1 axis increases proliferation of meningioma tumor cells

**DOI:** 10.1093/noajnl/vdaa008

**Published:** 2020-01-21

**Authors:** Sara Ferluga, Daniele Baiz, David A Hilton, Claire L Adams, Emanuela Ercolano, Jemma Dunn, Kayleigh Bassiri, Kathreena M Kurian, Clemens O Hanemann

**Affiliations:** 1 Faculty of Health: Medicine, Dentistry and Human Sciences, Institute of Translational and Stratified Medicine, University of Plymouth, Plymouth, UK; 2 Cellular and Anatomical Pathology, Plymouth Hospitals NHS Trust, Plymouth, UK; 3 Department of Neuropathology, Pathology Sciences, Southmead Hospital, Bristol, UK

**Keywords:** brain, cancer, EGFR, meningioma, STAT1

## Abstract

**Background:**

Meningiomas are the most frequent primary brain tumors of the central nervous system. The standard of treatment is surgery and radiotherapy, but effective pharmacological options are not available yet. The well-characterized genetic background stratifies these tumors in several subgroups, thus increasing diversification. We identified epidermal growth factor receptor–signal transducer and activator of transcription 1 (EGFR–STAT1) overexpression and activation as a common identifier of these tumors.

**Methods:**

We analyzed STAT1 overexpression and phosphorylation in 131 meningiomas of different grades and locations by utilizing several techniques, including Western blots, qPCR, and immunocytochemistry. We also silenced and overexpressed wild-type and mutant forms of the gene to assess its biological function and its network. Results were further validated by drug testing.

**Results:**

STAT1 was found widely overexpressed in meningioma but not in the corresponding healthy controls. The protein showed constitutive phosphorylation not dependent on the JAK–STAT pathway. *STAT1* knockdown resulted in a significant reduction of cellular proliferation and deactivation of AKT and ERK1/2. STAT1 is known to be activated by EGFR, so we investigated the tyrosine kinase and found that EGFR was also constitutively phosphorylated in meningioma and was responsible for the aberrant phosphorylation of STAT1. The pharmaceutical inhibition of EGFR caused a significant reduction in cellular proliferation and of overall levels of cyclin D1, pAKT, and pERK1/2.

**Conclusions:**

STAT1–EGFR-dependent constitutive phosphorylation is responsible for a positive feedback loop that causes its own overexpression and consequently an increased proliferation of the tumor cells. These findings provide the rationale for further studies aiming to identify effective therapeutic options in meningioma.

**Key Points**

Meningiomas are the most common primary brain tumor.
We show STAT1 is overexpressed and constitutively activated dependent on EGFR activation leading to increased proliferation.
EGFR inhibition with newer inhibitors reduces tumor cell proliferation.

**Importance of the Study**

Meningioma accounts for 37% of primary brain tumors. This year in the United States an estimated 32 000 people will be diagnosed with meningioma. These tumors can cause mild to severe morbidity and even WHO grade I can have a more aggressive clinical course. Therapeutic options are still limited to surgical resection and radiotherapy since more effort is needed to decipher the communal molecular mechanisms that define meningiomas despite their genetic background. Aiming to discover novel therapeutic targets, we identified STAT1 as aberrantly overexpressed and constitutively activated in most of the meningiomas examined. Its activation is dependent on the constitutive phosphorylation of EGFR and leads to increased proliferation of tumor cells. We show that specific EGFR inhibition can reduce tumor cell proliferation and we show evidence why previous trials failed. Therefore, we suggest that this therapeutic strategy be re-evaluated.

Meningiomas are the most common primary brain tumors, classified meningiomas as Grade I (~80%), atypical Grade II (15–20%), and anaplastic/malignant Grade III (1–3%). Surgery is the primary choice of treatment; complete resection may be curative but it can be achieved only for permissive locations.^1^ The genetic background of meningioma is well characterized, with inactivation/deletion of *NF2* found in ~60% of sporadic meningiomas.^2^

Previously, we identified phosphorylated signal transducer and activator of transcription 1 (STAT1) as overexpressed in the grade I meningioma cell line^3^ and phosphorylated STAT1 in meningioma tissue of all grades.^4^ In addition, we identified the phosphorylation of STAT3 among remaining STAT family members.^3,4^ STAT1 belongs to the STAT protein family that comprises 7 members (STAT1–4, STAT5A, STAT5B, and STAT6), and it can be phosphorylated on the tyrosine 701 (Y701) and the serine 727 (S727).^5,6^ STATs are essential components of the evolutionarily conserved JAK–STAT signaling pathway^4,7^ that plays a role in immune response^8,9^ and its dysregulation is linked to cancer.^10,11^ This canonical pathway is activated by ligands including interferons, interleukins, and some growth factors, binding to their receptors thus inducing phosphorylation of the Janus kinases (JAKs), leading to tyrosine-STAT phosphorylation by JAKs.^4,6^ In addition STATs can also be phosphorylated by receptor tyrosine kinases and cytoplasmic non-receptor tyrosine kinases.^5^ Phosphorylated STATs homo- and heterodimerize entering the nucleus to regulate transcription of target genes.^6,12^ JAKs include JAK1–3 and TYK2. JAK1 and JAK2 are phosphorylated following type-II interferon (IFNγ) stimulation, while JAK1 and TYK2 are activated in type-I interferon signaling (IFNα, IFNβ, etc.).^4–6^ Activated JAK–STAT pathway can be quenched by the suppressors of cytokine signaling (SOCSs), the protein inhibitors of activated STAT (PIASs), and the protein tyrosine phosphatases (PTPs).^5^

Activated STAT1 acts as a transcriptional regulator, controlling its own transcription as well as the expression of several IFN-regulated genes.^13,14^ STAT1 was considered a tumor suppressor as its expression correlated with good prognosis in several types of cancer.^15–18^ However, other studies established a pro-tumorigenic role of STAT1, which correlated with its overexpression and activation.^19^ Due to its function in sensing and regulating cytokine production, STAT1 exerts a role in promoting an immunosuppressive tumor environment.^19,20^ Hence, the overall role of STAT1 in cancer remains complex suggesting that its function is most likely cancer type-dependent.

In the present study, we identified STAT1 as overexpressed and phosphorylated in meningioma compared to normal and we show that its overexpression correlates with an increased proliferation of the tumor cells as well as activation of AKT and ERK1/2. We demonstrate that STAT1 overexpression and phosphorylation is not dependent on the JAK–STAT pathway but it depends on a positive feedback loop caused by the constitutive activation of the epidermal growth factor receptor (EGFR). The pharmaceutical inhibition of EGFR in meningioma caused the deactivation of STAT1 and other cancer-related pathways, eventually leading to a significant reduction in cellular proliferation.

Our findings underline a crucial role of the EGFR and STAT1 signaling in the pathology of meningiomas and point to a therapeutic potential of its inhibition.

## Materials and Methods

### Meningioma Specimens, Tumor Digestion, and Primary Meningioma Cultures

Meningioma specimens were collected following the ethical approvals received a unique MN number (Supplementary Table S1). Normal meningeal tissue (NMT) was purchased from Analytical Biological Service, Inc.

Primary cells were generated from 36 fresh tumor tissues. Tissues were disaggregated in DMEM with 15% FBS, 100 U/ml penicillin/streptomycin, and 20 U/ml Collagenase III (Worthington Biochemical Corp.) for 2 h at 37°C; after cells were pelleted at 1000 rpm for 5 min, resuspended and seeded (modified from ref. ^21^). MN cells were cultured in DMEM at 37°C in 5% CO_2_. Human meningeal cells (HMCs) (Caltag Medsystems Ltd) were grown in the recommended medium at 37°C in 5% CO_2_. Cells were kept on average 4–5 passages.

Normal HMCs were purchased from ScienCell (UK distributor: Caltag Medsystems; Catalog # 1400), U251 glioma cells were purchased from ECACC (Catalog # 09063001), an immortalized grade 1 meningioma cell line BM-1 was from DSMZ (Catalog # ACC 599) and authenticated via genomic fingerprinting (Eurofins Genomics Europe Applied Genomics GmbH).

### Western blotting, immunofluorescence, and immunohistochemistry

Western blots (WBs) from 26 frozen tissues and cell cultures were performed as previously described.^3^ All primary antibodies used are listed in Supplementary Table S2. Immunoreactive bands were quantified using Scion Image software and each band was normalized versus the corresponding GAPDH.

Immunofluorescence of 38 paraffin-embedded tissues was performed as previously described.^3^ Confocal microscopy was executed using a Leica DMI6000B; Z-stack micrographs were taken using the 40× or 63× objectives. Immunofluorescent images for STAT1-silencing studies were taken with the Olympus CKX41 with the 20× objective; images were processed with the QCapture Pro 6.0 software.

For immunohistochemistry, paraffin sections (4 μm) were processed as described.^22^ Avidin–biotin blocking solution was used with EDTA pretreatment. Sections were incubated with appropriate biotin-labeled secondary antibody and with horseradish peroxidase for detection using Vectashield Elite (Vector Laboratories UK) according to the manufacturer’s protocol. As a control, sections were incubated with the omission of the primary antibody.

Results were reviewed “blind” to the histological grade by a neuropathologist (DAH). Semiquantitative assessment of the intensity of immunoreactivity was undertaken and scored as follows: 0, none; 1, weak; 2, moderate; 3, strong.

### RNA Isolation and Gene Expression Analysis

Total RNA was extracted from 95 frozen tissues and cells using the Qiazol reagent (Qiagen UK), following the manufacturer’s protocol. The quality, integrity, and concentration of RNA were established using the NanoDrop ND-2000 (Thermo Fisher Scientific UK).

Real-time PCR (qPCR) was conducted using 50 ng/well employing the EXPRESS One-Step SYBR GreenER kits (Invitrogen) on a LightCycler 480 System (Roche Diagnostics), following the manufacturer’s protocol (primers annealing temperature = 58°C). Primers used were PrimePCR SYBR Green Assay STAT1 (BioRad), hGAPDH (2 µM, Invitrogen; Forward: 5′-GAGAAGGCTGGGGCTCATTT-3′; Reverse 5′-AGTGATGGCATGGACTGTGG-3′). Relative gene expression analysis of STAT1 and GAPDH was calculated using the 2^−ΔΔCt^ method,^23^ employing the HMC as calibrator.

### STAT1 Silencing and Overexpression

STAT1 shRNA Lentiviral Particles (Santa Cruz Biotechnology, sc-44123-V), containing 3 target-specific constructs that encode 19–25 nt (plus hairpin) or scramble shRNA control (Santa Cruz Biotechnology, sc-108080), were added onto the cells in media containing protamine sulfate salt (8 μg/ml) (Sigma). Cells were infected for 48 h before applying puromycin (5 μg/ml) for 3 days.

*STAT1-WT* gene was cloned into pcDNA3.1+ in a two-step process using the following primers: STAT1-F1 (5′-AAAGCTAGCGGCCGGCCATGTCTCAG-3′), STAT1-R1 (5′-CGTCTCGAGGTCAATTACCAAACCAGGCT-3′) for the first part; STAT1-2F (5′-GACCTCGAGACGACCTCTCT), STAT1-2R (5′-AGTGTTTAAACTTAATTAACTATACTGTGTTCA-3′) for the second part. The 551 bp long STAT1 part in between the restriction sites *Hind*III and *Eco*RI was synthesized (GeneArt, Thermo Fisher Scientific) to generate the following mutations: Y701F, S727E, and Y701F/S727E; each one was cloned into pcDNA-STAT1-WT to replace the wild-type part. All generated plasmids were sequenced before further use (Eurofins). U251-MG cells were transfected and selected as previously described.^24^

### Ki-67 Staining and Proliferation Assay

For Ki-67 staining, cells were grown on chamber slides, lentivirus-transfected and stained as previously described.^3^

For U251-MG proliferation assay, the pool of U251-MG selected cells, transfected with pcDNA, STAT1-WT, and the 3 mutants, were seeded at 1000 cells/well in 96-well plates and proliferation was determined after 24, 48, and 72 h using the “CellTiter-Glo Luminescent Cell Viability Assay” as recommended by the supplier (Promega).

For drug testing, meningioma cells (~3000 cells/well) were plated in 96-well culture plates and allowed to proliferate for 24 h. Cell proliferation was calculated as a percentage of control cells. Graphs were generated using GraphPad Prism 5.

### Flow Cytometry Analysis

Confluent meningioma cells were resuspended in ice-cold staining buffer (PBS, 2% FBS) at a final concentration of 1 × 10^5^ cells. Cells were stained for 30 min at RT in the dark with the following: CD45-FITC, HLA-DR-PE, CD14-PerCP5.5, and CD44—APC (Becton Dickinson Biosciences, Pharmingen), washed twice with 2 ml of staining buffer and centrifuged at 1500 rpm for 5 min at 4°C. The relevant single isotype controls were used. Data acquisition was collected on 1 × 10^4^ cells on an Accuri flow cytometer (BD Biosciences) and analysis was performed using Flow Jo software v10.0 (FlowJo LLC).

### Statistical Analysis

Probability (*P*) values were calculated using the Student’s *t*-test or the one-way analysis of variance, using GraphPad Prism 5.01 and MS Excel 2016 software. *P*-values <.05 were considered statistically significant. The results are expressed as means ± SD or ± SEM.

## Results

### STAT1 Is Overexpressed and Aberrantly Activated in Meningioma

**Figure 1.**
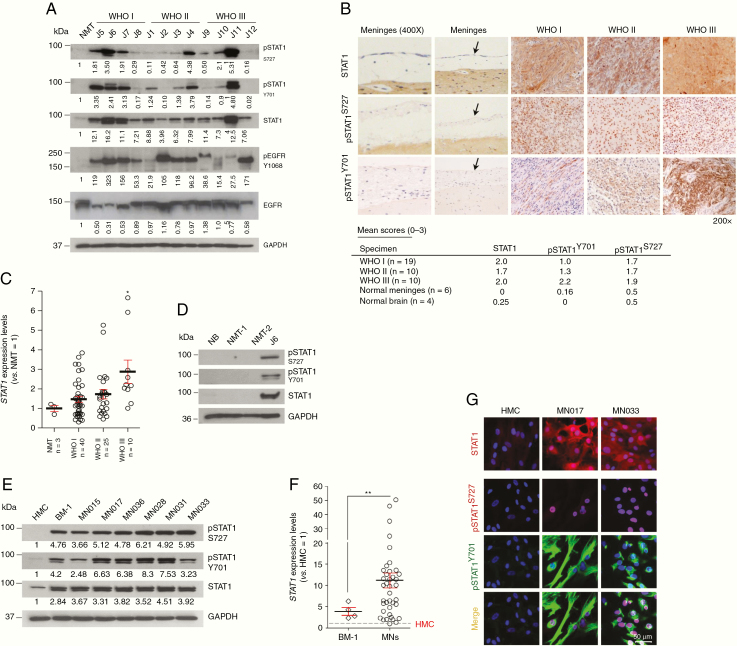
STAT1 and its phosphorylated forms are overexpressed in meningioma. (A) Representative WB analysis showing the expression of total and pSTAT1 in different grade meningiomas versus normal meningeal tissue (NMT). (B) Representative images showing the IHC staining of STAT1 and pSTAT1 in the 3 grades meningiomas compared to normal meninges (see black arrows) at 200× magnification. Mean scores are presented in the table below for the specimens and the normal controls examined (see also Supplementary Table S1 for the full list of specimens examined and the corresponding scores—*n* = 47). (C) *STAT1* expression levels in WHO I (*n* = 40), WHO II (*n* = 25), and WHO III (*n* = 10) meningioma tumors normalized versus NMT. Data are presented as mean ± SEM; *P ≤ .05. (D) WB showing pSTAT1 and STAT1 in normal brain (NB) and additional normal meninges (NMT-1 and NMT-2) compared to sample J6 (meningioma) as a positive control. (E) Representative WB analysis of STAT1 and pSTAT1 in BM-1 and in WHO I MN cells (MNs) versus HMC. (F) *STAT1* expression levels in BM-1 (*n* = 4) and in MN cells (*n* = 24) normalized versus HMC. Data are presented as mean ± SEM; **P ≤ .01. (G) Confocal z-stack images showing the immunofluorescent staining of STAT1 (red) and pSTAT1 (Y701, green and S727, red) in MN cells versus HMC. Scale bar 50 μm. Nuclei were stained with DAPI (blue).

We analyzed STAT1 expression in meningioma tumors compared to normal meninges (NMT). In all cases STAT1 was overexpressed and in most of the cases, we detected high levels of phosphorylated STAT1 (Y701 and S727) (representative WB of Figure 1A and qPCR of Figure 1C). Immunohistochemical studies validated STAT1 overexpression in all meningioma samples (Figure 1B); also pSTAT1-Y701 and -S727 showed higher staining compared to normal meninges and an increasing score throughout the grades. As a control, we further analyzed STAT1 and pSTAT1 abundance in 2 additional normal meninges and a normal brain (Figure 1D).

Then, we examined STAT1 expression and phosphorylation in meningioma-derived primary cells (MN) and BM-1^25^ compared to HMC. MN cells were used between passage 3 and 5 and no B/T lymphocytes or infiltrating macrophages were detected (Supplementary Figure S1A). All cells were vimentin-positive^26^ and CD90-negative, suggesting no fibroblasts contamination^27^ (Supplementary Figure S1B). STAT1 was found overexpressed in BM-1 and MNs compared to HMC and both pSTAT1-Y701 and -S727 were present across all samples while faint and undetectable in HMC (Figure 1C). Quantitative PCR analysis confirmed that *STAT1* expression was higher in most of the MNs and in BM-1 compared to control (Figure 1F). Of note, STAT1 overexpression was independent of Merlin status (Supplementary Figure S1C and D).

Furthermore, pSTAT1-Y701 showed a cytoplasmic localization while pSTAT1-S727 was nuclear (Figure 1B), in agreement with the immunofluorescent staining of primary MN cells (Figure 1G).

Overall, we examined 131 meningiomas versus 10 normal meninges and 5 normal brains and we demonstrate substantial overexpression of STAT1 in 100 of them with a variety of methods (Supplementary Table S1).

### STAT1 Constitutive Phosphorylation Is Not Dependent on the JAK–STAT Pathway

**Figure 2.**
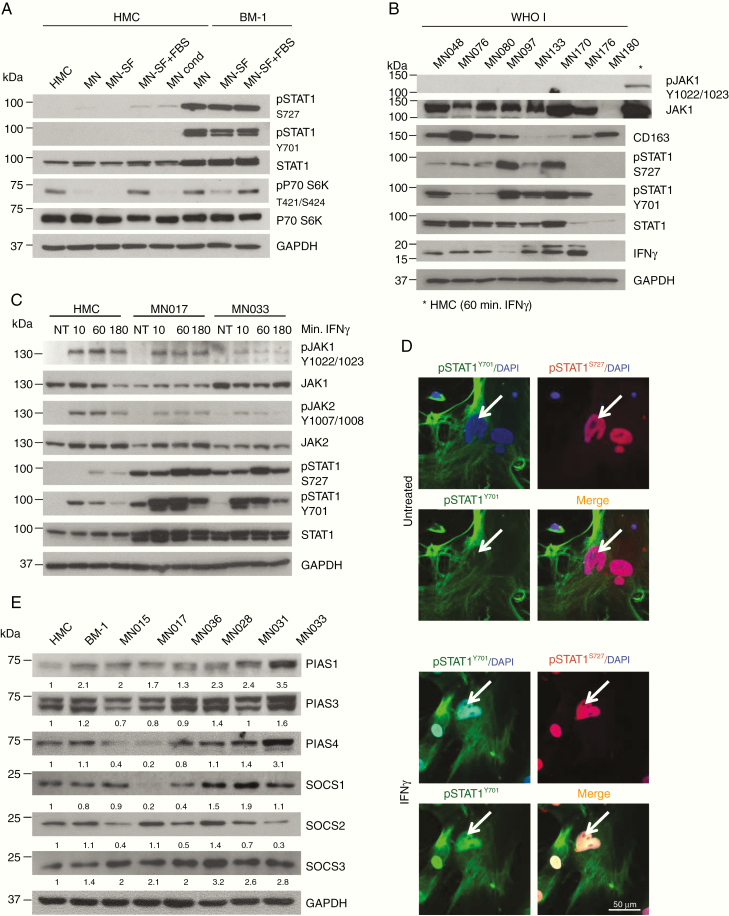
STAT1 phosphorylation in meningioma cells is not dependent on the JAK–STAT pathway. (A) WB of WHO I meningioma tumor tissue lysates (*n* = 8); the presence of gamma interferon (IFNγ) and macrophage infiltration (CD163) into the tumor were analyzed in relation to STAT1 and pSTAT1 levels. Phospho-JAK1 was used to detect activation of the JAK–STAT pathway (*positive control for pJAK1 antibody). (B) WB of total and pSTAT1 in BM-1 and HMC cells, grown in different culture condition. HMC, HMC cells media; MN, MN cells media; MN-SF, MN-serum free media; MN-SF+FBS, MN serum free for 24 h + FBS for 24 h; MN Cond, meningioma cells-conditioned media. (C) WB analysis of STAT1 and pSTAT1 protein levels in HMC and 2 primary MN cells after IFNγ treatment at the concentration of 50 ng/ml for the indicated amount of time. Phosho-JAK1 and pJAK2 are shown to confirm the activation of the JAK–STAT pathway. (D) Representative confocal images (z-stack) showing localization of pSTAT1-Y701 (green) and pSTAT1-S727 (red) in primary MN cells before and after IFNγ stimulation (50 ng/ml for 1 h). Scale bar 50 μm. Nuclei were stain with DAPI (blue). (E) WB analysis of SOCSs and PIASs protein levels in BM-1 and primary MNs compared to HMC.

To further investigate STAT1 phosphorylation in the context of the tumor environment, we examined meningioma tumor lysates for the presence of interferon gamma (IFNγ) and tumor-associated macrophages by using CD163 marker staining preferentially M2 macrophages.^28^ Variable protein levels of IFNγ and CD163 were detected, but there was no evident correlation with STAT1 phosphorylation and no JAK1 phosphorylation was detected (Figure 2A).

STAT1 usually becomes phosphorylated as a result of JAK–STAT pathway activation in response to external stimuli.^6^ We examined whether STAT1 overexpression and phosphorylation was dependent on the culture conditions and secreted factors. Culturing HMC in serum-free (SF) media and in BM-1 conditioned media, and BM-1 in SF media, we confirmed that STAT1 overexpression and phosphorylation was not due to external factors, but most likely to an intrinsic activation (Figure 2B).

Next, we decided to test the ability of the JAK–STAT pathway to respond to activating stimuli in meningioma cells. HMC and 2 MNs were treated with IFNγ; in HMC, JAK1 and JAK2 activated within 10 min after treatment as well as pSTAT1-Y701 while pSTAT1-S727 phosphorylated within 1 h. The same behavior was observed in MNs confirming that the JAK–STAT pathway was functional; however, STAT1 was constitutively phosphorylated in nontreated cells while pJAK1 and pJAK2 were not (Figure 2C). The same experiment, performed using interferon alpha (IFNα), produced comparable results (Supplementary Figure S2A).

After activation, pSTAT1 is known to dimerize and translocate into the nucleus.^6^ IFNγ treatment was indeed able to induce pSTAT1-Y701 nuclear internalization (Figure 2D; Supplementary Figure S2B). Thus, the JAK–STAT1 pathway can be activated via IFN in meningioma cells but there was also an IFN-independent intrinsic activation.

STAT1 constitutive phosphorylations could be due to a deficient deactivation of the pathway.^4,5,29^ Thus, we analyzed the levels of the SOCSs and the PIASs in HMC, BM-1, and MN cells (Figure 2E), which did not correlate with the constitutive phosphorylation of STAT1 observed in these samples (Figure 1E).

Overall, these data suggest that the JAK–STAT pathway is functional but not over-activated. Therefore, we hypothesized other mechanisms must be involved in maintaining STAT1 in a constitutive phosphorylated form in the meningioma samples analyzed.

### STAT1 Overexpression Is Associated With an Increased Proliferation of Meningioma Cells

**Figure 3.**
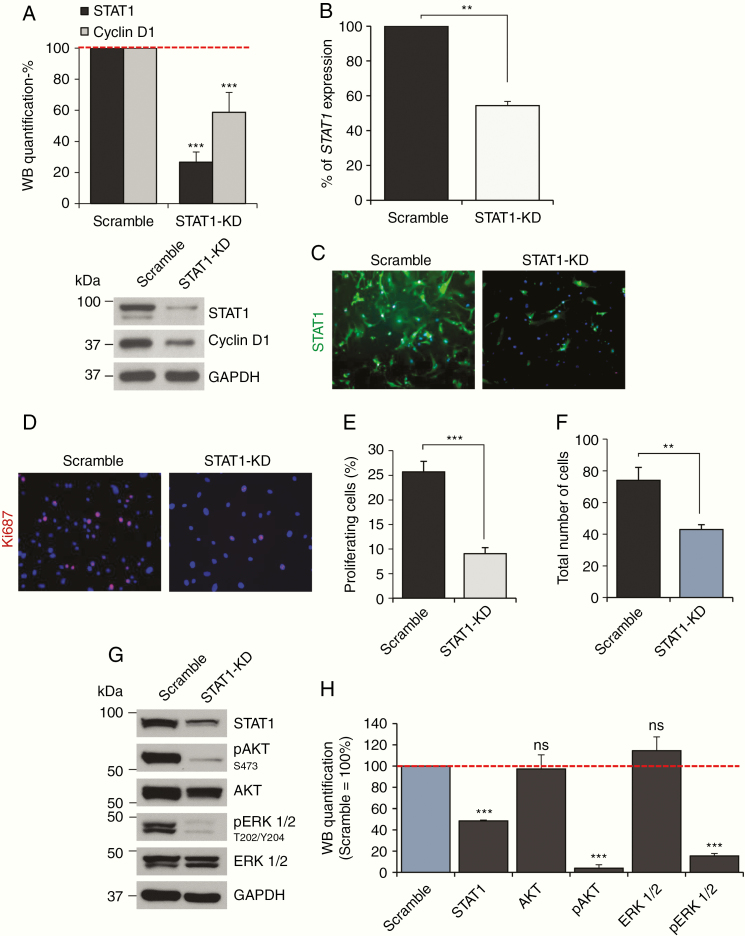
STAT1 overexpression increases meningioma cells proliferation. (A) Histogram representing the percentage of statistical reduction in STAT1 and cyclin D1 protein levels after *STAT1* shRNA-mediated silencing using a pool of 3 shRNAs in 3 primary MN cells compared to scramble; a representative WB is shown underneath. Data are presented as mean ± SD; ***P ≤ .001. (B) Percentage of reduction in *STAT1* expression associated with *STAT1* shRNA-mediated silencing compared to control shown in (A). Data are presented as mean ± SEM; **P ≤ .01. (C and D) Representative images of the immunofluorescent staining of STAT1 (green) and the proliferation marker Ki-67 (red) (D) after *STAT1* shRNA-mediated silencing compared to scramble. Nuclei are stained with DAPI (blue). (E and F) Histogram presenting the statistical reduction of proliferating cells and a total number of cells (F) after STAT1-KD compared to control. Data are presented as mean ± SD; ***P ≤ .001, **P ≤ .01. (G) Representative WB, showing the reduction in AKT and ERK1/2 phosphorylation following STAT1 silencing. (H) Histogram representing the WB quantification of total and phosphorylated AKT and ERK1/2 following STAT1 silencing in 3 primary MN cells, ***P ≤ .001; ns, not significant.

To investigate the biological significance of STAT1 overexpression in meningioma we silenced the protein in MN cells. Lentiviral-mediated shRNA delivery into the cells produced a more than 70% reduction in protein expression (Figure 3A) and a 50% reduction in gene expression levels compared to scramble (Figure 3B). STAT1-silenced cells displayed a reduction in STAT1 immunofluorescent staining as well as a reduction in Ki-67-positive cells (Figure 3C). Proliferating cells were reduced from ~22% to less than 5% in MNs (Figure 3D and E). This was in agreement with the reduction of the total number of cells (Figure 3F) and a 40% reduction of cyclin D1 (Figure 3A). A similar effect was observed in BM-1 cells (Supplementary Figure S3A–D). Taken together, our results demonstrate that STAT1 overexpression is associated with an increased proliferation of meningioma tumor cells.

The MAPK–ERK and the AKT pathways are known to be active in meningioma and to influence tumor progression.^30^ After STAT1-KD, both AKT and ERK1/2 showed a 95% and 80% reduction in protein phosphorylation, respectively (Figure 3G and H), supporting a critical involvement of STAT1 in the activation of pro-proliferative pathways.

### Phosphorylated STAT1 Affects Activation of AKT and ERK1/2 and Cellular Proliferation

**Figure 4.**
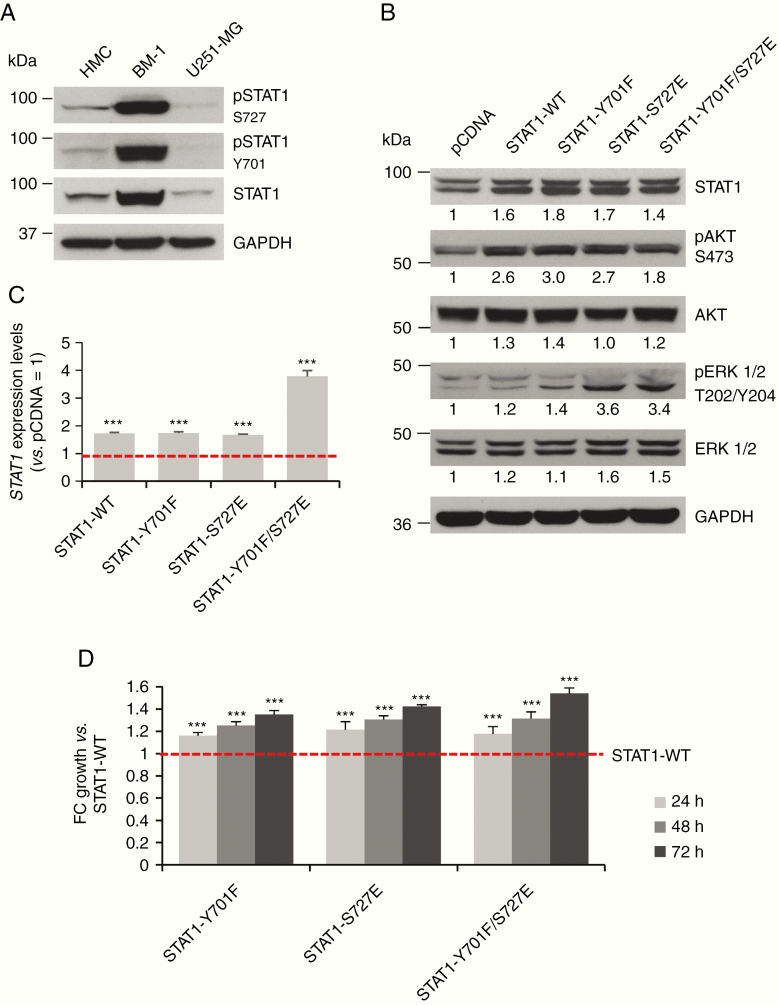
STAT1 activating mutations induce phosphorylation of AKT, ERK1/2 and increased proliferation of U251-MG cells. (A) WB representing total and phosphorylated STAT1 levels in U251-MG compared to HMC and BM-1 cells. (B) WB showing overexpression of STAT1-WT and activating mutants in U251-MG cells and the related activation of pAKT and pERK1/2. (C) *STAT1* expression levels in U251-MG cells normalized versus *STAT1* expression levels in pcDNA transfected cells. Data are presented as mean ± SEM; ***P ≤ .001. (D) Histogram presenting the statistical increase in cell proliferation in U251-MG cells overexpressing the activating STAT1 mutants (STAT1-Y701F, STAT1-S727E, STAT1-Y701F/S727E). Data were normalized for STAT1-pcDNA-transfected cells and presented as FC of growth versus STAT1-WT; ***P ≤ .001.

We used phosphomimetics to further characterize the effects of STAT1 phosphorylation. Phenylalanine (F) and glutamic acid (E) are used to mimic the structure of a phosphorylated tyrosine (Y) and phosphorylated serine (S), respectively.^31^ We produced 3 different STAT1 mutants: Y701F, S727E, and the double mutant Y701F/S727E. Since STAT1 is constitutively phosphorylated in meningioma, we used U251-MG cells as a model because this cell line showed levels of total and pSTAT1 lower than HMC (Figure 4A). STAT1 overexpression in U251-MG for wild-type (WT) and mutants was confirmed by WB and qPCR (Figure 4B and C). STAT1 overexpression in U251-MG cells determined increased phosphorylation of AKT and ERK1/2, where the effect was particularly evident for pERK1/2 in STAT1-S727E and STAT1-Y701F/S727E mutants (Figure 4B).

The proliferation of transfected cells was measured over a period of 72 h and normalized for the empty-vector control. All STAT1 mutants showed a significantly increased proliferation rate compared to STAT1-WT; interestingly, the double mutant STAT1-Y701F/S727E, which represents STAT1 in its maximal activated condition, determined the highest pro-proliferative effect in U251-MG cells (Figure 4B and D).

These experiments confirmed that the constitutive phosphorylation of STAT1 on both phosphosites affects the activation of the AKT and ERK1/2 pathways as well as the proliferation of the cells in agreement with STAT1 knockdown results in meningioma.

### EGFR Constitutive Phosphorylation Is Responsible for STAT1 Overexpression and Activation

**Figure 5.**
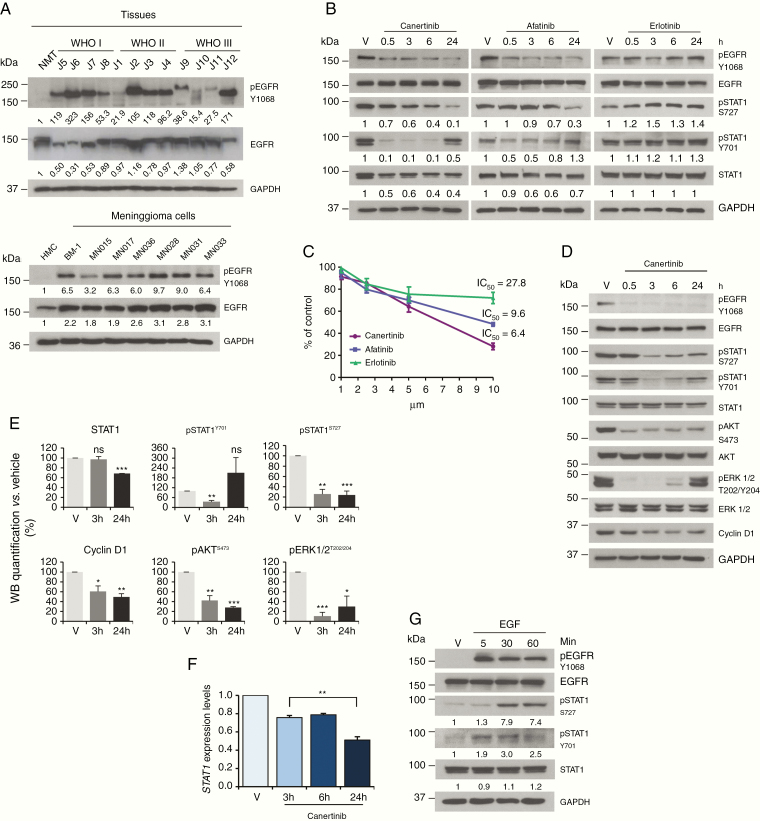
The constitutive activation of the EGFR in meningioma induces STAT1 phosphorylation. (A) Representative WB analysis of total and pEGFR-Y1068 in meningioma, when compared to control. Upper panel: WHO I, II, and III meningioma tissues compared to NMT; lower panel: BM-1 and primary MN cells compared to HMC. (B) WB of STAT1 and pSTAT1 protein levels after treatment with 5 μM of canertinib, afatinib, and erlotinib in BM-1 cells. The reduced levels of pEGFR-Y1068 confirmed drug activity. (C) ATP-proliferation assay performed in BM-1 cells after treatment with different concentrations of canertinib, afatinib, and erlotinib for 24 h. (D) WB analysis of STAT1, pSTAT1, and other markers of proliferation in primary MN cells after treatment with 10 μM of canertinib. (E) Histograms representing WB quantification at 3 and 24 h for STAT1, pSTAT1, pAKT, pERK1/2, and cyclin D1 after canertinib treatment in 3 different primary MN cells (see Supplementary Figure S4). Data are presented as mean ± SEM, *P < .05, **P < .01, ***P < .001. (F) qPCR analysis showing the statistical reduction of *STAT1* gene expression at 3, 6, and 24 h after treatment with 10 μM of canertinib (*n* = 3). Data are presented as mean ± SEM; **P < .01. (G) WB representing STAT1 and pSTAT1 in BM-1 cells, following treatment with EGF (50 ng/ml) for 5, 30, and 60 min.

It has been previously shown that STAT1 can be phosphorylated by EGFR, a key tyrosine kinase relevant to the majority of tumors.^32,33^ We examined the EGFR status in meningioma tissues and cells, detecting high levels of pEGFR in both tumor lysates and meningioma cells, when compared to NMT and HMC (Figure 5A).

To test whether the constitutive phosphorylation of EGFR was responsible for STAT1 phosphorylation, we treated BM-1 cells with 3 different EGFR inhibitors, canertinib and afatinib (second-generation irreversible inhibitors) and erlotinib (first-generation reversible inhibitor), for 30 min, 3, 6, and 24 h.^34^ Canertinib (and similarly afatinib) decreased STAT1 expression of about 60% within 24 h; pSTAT1-Y701 was almost abolished 30 min after treatment but was restored at 24 h while pSTAT1-S727 showed a decrease of about 90% compared to vehicle at 24 h (Figure 5B). Almost no effect on total and pSTAT1 was detected after treatment with erlotinib, which did not cause an evident decrease in pEGFR-Y1068 after treatment (Figure 5B).

EGFR blockade via canertinib and afatinib decreased pSTAT1 levels and determined a concentration-dependent decrease of cellular proliferation already at 24 h after treatment (Figure 5C), with erlotinib being ineffective.

Since canertinib showed the strongest effect on STAT1 in BM-1 cells, we tested its effects on primary MNs (Figure 5D). Canertinib was active in reducing EGFR constitutive phosphorylation in MN cells, reducing pSTAT1 levels after canertinib treatment; pSTAT1-S727 reduced of 65% already 3 h after treatment and stayed low over the 24 h; phosphorylated STAT1-Y701 also showed about 50% reduction 3 h after treatment and recovered between 6 and 24 h (Figure 5D and E; Supplementary Figure S4).

Phospho-AKT and pERK1/2 showed a decrease of about 70% and cyclin D1 reduced to 50% in 24 h (Figure 5D and E; Supplementary Figure S4).

We wanted to examine whether the inhibition of pEGFR and thus of pSTAT1 had any effect on *STAT1* expression, as STAT1 is known to regulate its own transcription.^35^*STAT1* expression levels reduced by ~50% 24 h after treatment with canertinib in MNs (Figure 5F), consistently with a 30% reduction in protein level observed by WB analysis (Figure 5D and E; Supplementary Figure S4).

Lastly, to confirm the link between EGFR activation and STAT1 phosphorylation, we treated BM-1 cells with the epidermal growth factor (EGF) for 5, 30, and 60 min. Upon EGF treatment STAT1 was phosphorylated on Y701 within 5 min and on S727 within 30 min (Figure 5G).

Hence, we showed that EGFR is responsible for STAT1 overexpression and constitutive activation in meningioma, which consequently increases the proliferation of the tumor cells.

## Discussion

Meningiomas are the most common primary brain tumor but there are no therapeutic options available other than surgery and radiotherapy.^1,36^ The well-defined genetic background of meningioma is leading toward an increasing stratification of these tumors into subtypes^37,38^; however, common features should still be investigated.

We identified STAT1 as overexpressed and activated in 84% of meningioma examined. The only study exploring the expression levels of STAT and JAK superfamilies in meningiomas was published in 1999 showing higher immunoreactivity of JAK1 (see also Supplementary Figure S2C), JAK2 and the STATs in meningiomas compared to normal dura.^39^ Our data confirmed the expression of the JAKs in MN cells and HMCs; we showed that the JAK–STAT pathway is activated by IFNα and IFNγ, inducing nuclear localization of pSTAT1 as seen before.^39^ As previously reported,^40^ activation of STAT1 after INFγ stimulation occurs via JAKs by phosphorylation on Y701, resulting in pSTAT1 translocation into the nucleus and subsequent phosphorylation at S727.^41^ Double phosphorylation is required for maximal STAT1 activity. However, we show that STAT1 is constitutively phosphorylated in MNs but not in HMC, even without IFN stimulation and in serum-free conditions. In tumor lysates, STAT1 phosphorylation was not consistent with the presence of M2-polarized macrophages or IFNγ suggesting that the constitutive activation of STAT1 was not related to the JAK–STAT pathway.

To better understand the meaning of this STAT1 phosphorylation we used phosphomimetics, generating STAT1-Y701F, STAT1-S727E, and STAT1-Y701F/S727E mutants. The overexpression of these mutants induced activation of 2 central nodes in cancer signaling, AKT and ERK1/2, and increased cellular proliferation. A similar approach was used on STAT3 in human prostate cancer cells, where the mutant STAT3-Y705F/S727E promoted survival, growth, and invasion. They showed that the mutation S727E was increasing the transcription of c-Myc, which is an essential activator of cell growth and proliferation.^31^ It is very likely that a similar mechanism is happening also in meningioma, where STAT1-S727 showed a predominant nuclear localization exerting its role of transcriptional regulator.

We also showed the link between STAT1 overexpression and the increased proliferation of the tumor cells. This effect is most likely linked to an activating cascade involving ERK1/2 and AKT, because their activated state and cell proliferation were almost aborted after STAT1 silencing. The activation of the MAPK pathway is involved in both proliferation and apoptosis in meningioma,^30^ and we recently published proteomic profiling of meningioma, identifying the aberrant activation of the PI3K–AKT pathway across all meningioma grades.^4^

Aiming to identify the kinase responsible for STAT1 activation, we examined the status of EGFR, a tyrosine kinase able to phosphorylate STAT1.^33,42,43^ EGFR was overexpressed and constitutively phosphorylated on Y1068 in all of the MN cells examined but not in HMC. To test whether EGFR phosphorylation was responsible for the constitutive activation of STAT1 we used 3 specific EGFR inhibitors canertinib, afatinib, and erlotinib.^44^ While canertinib and afatinib had a similar effect in reducing STAT1 phosphorylation on both phosphosites as well as on cell proliferation and viability, erlotinib did not produce any significant effect. Interestingly this result is consistent with the unsuccessful clinical trial of erlotinib on recurrent meningiomas.^45^ Erlotinib is a first-generation ATP-dependent reversible rather broad inhibitor.^46^ Afinitinib and canertinib are non-reversible second-generation inhibitors with high pEC_50_, https://www.proteomicsdb.org/#analytics/selectivity

In MN cells, canertinib (and afatinib) caused the de-phosphorylation of STAT1-Y701 and S727 within 6 and 24 h, respectively. Similarly, EGF stimulation induces an immediate and direct phosphorylation on Y701 and a later one on S727, suggesting the activation of an additional kinase downstream of EGFR, which is probably part of the MAPK–ERK1/2 pathway.^47^ Indeed previous studies in pancreatic cancer demonstrated the relationship between EGFR and the downstream signaling regulators like pAKT, pERK1/2, and cyclin D1.^33^ In agreement, after canertinib treatment and STAT1 silencing, we observed a significant reduction of pAKT and pERK1/2. Overall, levels of cyclin D1 also displayed a significant reduction, consistently with the reduction in proliferation observed after STAT1 silencing and canertinib treatment.

The observed reduction in STAT1 expression suggests a feedback regulatory mechanism of pSTAT1 on its own promoter, already documented,^35^ as well as an EGFR/HER2-dependent regulation as previously shown in glioblastoma and breast cancer cell lines.^48^

In conclusion, we provide clear evidence of STAT1 overexpression in meningioma of different genotype and its correlation with increased cellular proliferation. We demonstrate that STAT1 is aberrantly phosphorylated on both phosphosites, not because of the JAK–STAT pathway activation but because of the constitutive phosphorylation of EGFR, which elicits activation of the MAPK–ERK and PI3K–AKT pathways and an increase in the overall levels of cyclin D1 and STAT1. Although the whole mechanism should be additionally studied to give a thorough understanding of the activating cascade and all the partners involved in it, our studies set the basis for re-evaluating EGFR inhibition in meningioma as a possible therapeutic option.

## Supplementary Material

vdaa008_suppl_Supplementary_Figure_S1

vdaa008_suppl_Supplementary_Figure_S2

vdaa008_suppl_Supplementary_Figure_S3

vdaa008_suppl_Supplementary_Figure_S4

vdaa008_suppl_Supplementary_Table_S1

vdaa008_suppl_Supplementary_Table_S2

vdaa008_suppl_Supplementary_Table_and_Figure_Legend

## Notes

***Authorship Statement***. Designing and execution of most of the experiments, data interpretation, manuscript and figures preparation—S.F. Designing and execution of gene expression studies, data interpretation, writing of the related part, performing experimental revisions and addressing comments to reviewers, and proofreading of the manuscript—D.B. Designing and execution of immunohistochemistry, data interpretation, and writing of the related part—D.A.H. Designing and execution of the flow cytometry experiments, data interpretation, and writing of the related part—C.L.A. Managing of tumor digestions and primary MN cells cultures—E.E. Supporting with Western blot studies on MN Merlin status—J.D. Supporting with the initial identification of STAT1 in meningioma—K.B. Providing the majority of the samples involved in the study—K.M.K. Intellectual input to the critical design of the study, data interpretation, and manuscript preparation—C.O.H.
