# Supplementary figures and images for: Constitutive activation of the EGFR–STAT1 axis increases proliferation of meningioma tumor cells
Source: Neurooncol Adv. 2020 Jan 21;2(1):vdaa008. doi: 10.1093/noajnl/vdaa008 (PMC7212880; doi:10.1093/noajnl/vdaa008)

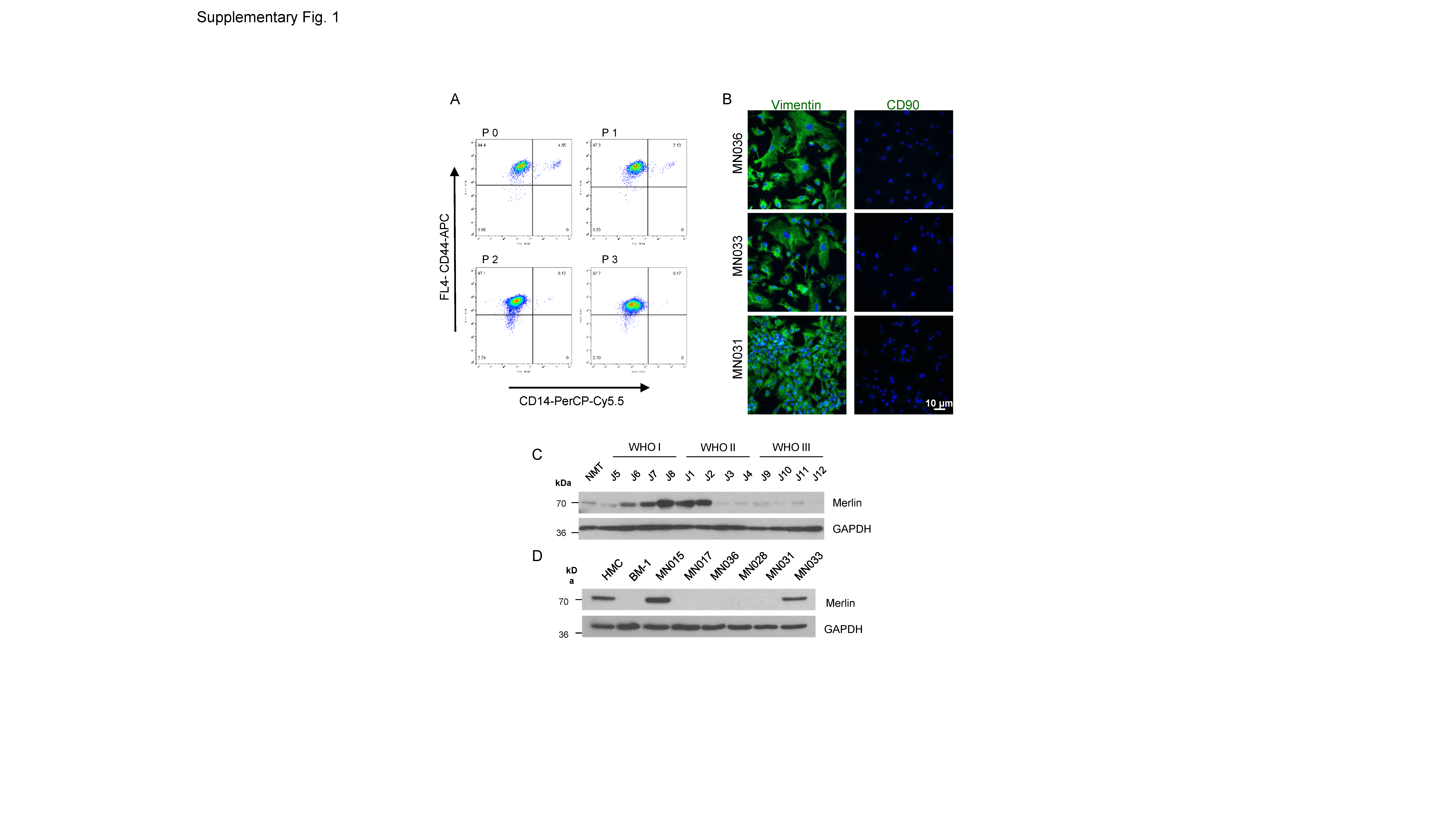

Supplement: vdaa008_suppl_Supplementary_Figure_S1 [file vdaa008_suppl_supplementary_figure_s1.png]

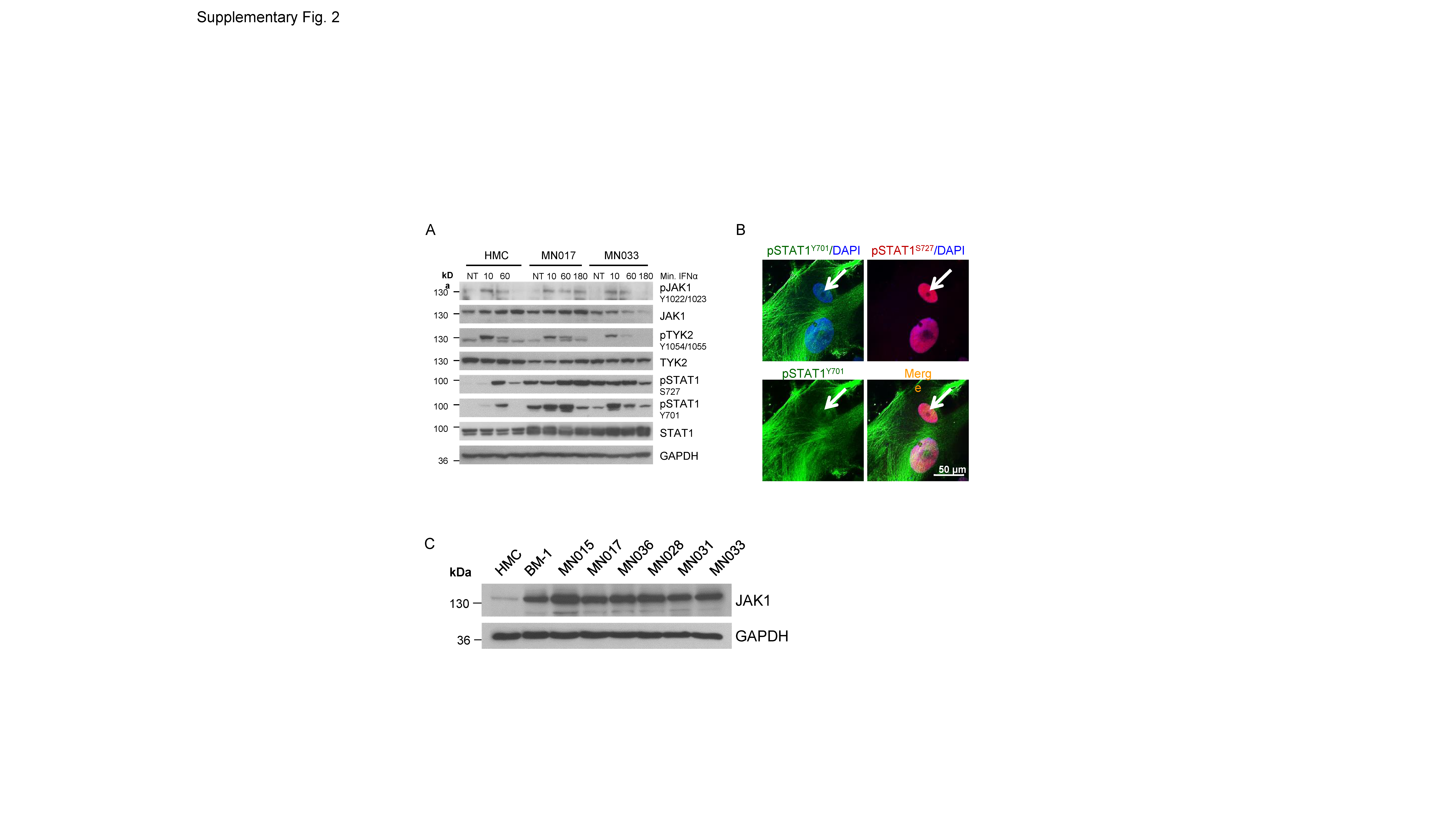

Supplement: vdaa008_suppl_Supplementary_Figure_S2 [file vdaa008_suppl_supplementary_figure_s2.png]

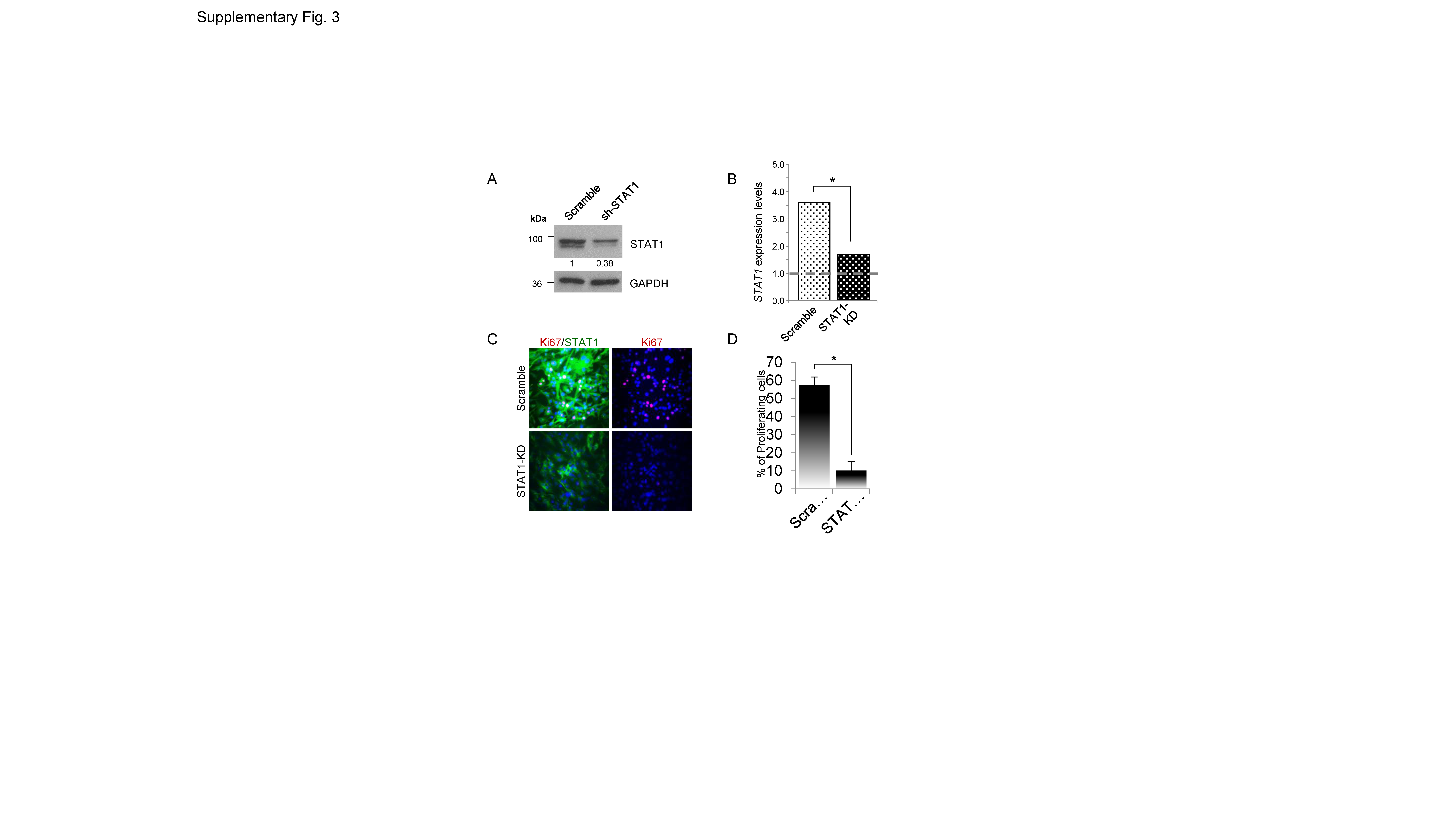

Supplement: vdaa008_suppl_Supplementary_Figure_S3 [file vdaa008_suppl_supplementary_figure_s3.png]

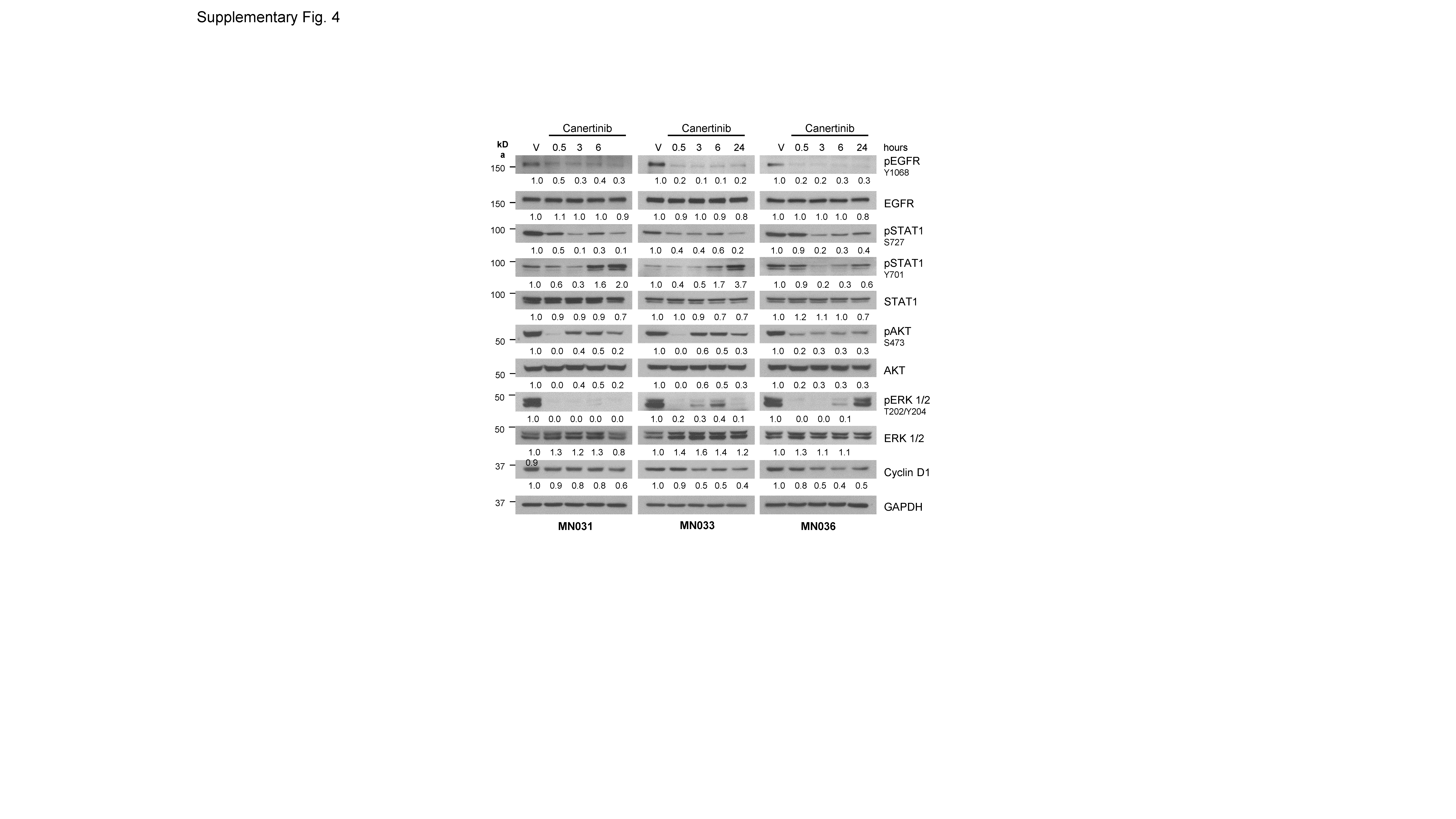

Supplement: vdaa008_suppl_Supplementary_Figure_S4 [file vdaa008_suppl_supplementary_figure_s4.png]
