# Supplementary material for: Constitutive activation of the EGFR–STAT1 axis increases proliferation of meningioma tumor cells
Source: Neurooncol Adv. 2020 Jan 21;2(1):vdaa008. doi: 10.1093/noajnl/vdaa008 (PMC7212880; doi:10.1093/noajnl/vdaa008)
Supplement: vdaa008_suppl_Supplementary_Table_S1 [file vdaa008_suppl_supplementary_table_s1.docx]

**Supplementary Table 1.** **Clinical cases examined in the study**. The table provide information about all the meningiomas tested and the level of STAT1 overexpression (gene expression for qPCR and protein expression for WB) detected *vs.* control., HMC=Human Meningeal Cells, L=left; R=right; n/a= not available; M=male; F=female, WB= Western Blot; qPCR= quantitative Polymerase Chain Reaction; IF= immunofluorescence; IHC= immunohistochemistry; Control (STAT1 expression = 1); ~ = STAT1 expression below 2; + = 2/3 times STAT1 overexpression; ++ = 5/6 times STAT1 overexpression; +++ = ≥ 10 times STAT1 overexpression.

| ID | Type, Location | WHO | | Gender | | Age of diagnosis | | Analysis | | STAT1 | |  |
| --- | --- | --- | --- | --- | --- | --- | --- | --- | --- | --- | --- | --- |
| Ben Men-1 cells | Benign meningioma cell line | | I | | F | | 68 | | WB, qPCR, IF | | + | |
| BTNW71 tissue | Anaplastic, L posterior fossa | | III | | F | | 70 | | qPCR | | ++ | |
| BTNW162 tissue | Anaplastic, L fronatl | | III | | F | | 76 | | qPCR | | ++ | |
| BTNW811 tissue | Anaplastic, L posterior fossa | | III | | F | | 64 | | qPCR | | ++ | |
| BTNW831 tissue | Anaplastic frontal | | III | | F | | 68 | | qPCR | | ++ | |
| BTNW1456 tissue | Anaplastic, L frontal | | III | | M | | 48 | | qPCR | | + | |
| MN001 tissue | Atypical, R frontal | | II | | M | | 50 | | IHC, qPCR | | + | |
| MN005 cells | Fibroblastic, L posterior fossa | | I | | F | | 59 | | IHC, qPCR | | ++ | |
| MN015 cells | Psammomatous, cervical | | I | | F | | 61 | | WB, qPCR | | + | |
| MN017 cells | Transitional, frontal convexity | | I | | F | | 51 | | WB, qPCR, IF | | + | |
| MN020 tissue | Atypical, R parietal | | II | | F | | 39 | | qPCR | | ~ | |
| MN023 cells | Meningothelial, L parietal convexity | | I | | M | | 63 | | WB, qPCR | | +++ | |
| MN028 cells | Transitional, cervical | | I | | F | | 63 | | WB, qPCR, IF | | ++ | |
| MN031 cells | Psammomatous, thoracic | | I | | F | | 72 | | WB, qPCR, IF | | ++ | |
| MN033 cells | Transitional, anterior skull base basecranial fossa | | I | | F | | 65 | | WB, qPCR, IF | | + | |
| MN036 cells | n/a, R CPA | | I | | F | | 51 | | WB, qPCR | | ++ | |
| MN038 cells | Transitional, L parietal | | I | | F | | 79 | | qPCR | | +++ | |
| MN045 tissue | Atypical, extra axial parietal | | II | | M | | n/a | | qPCR | | ~ | |
| MN048 cells | Fibroblastic, L parietal | | I | | F | | 57 | | WB, qPCR | | +++ | |
| MN052 cells | Psammomatous, R frontal parafalcine | | I | | F | | 70 | | qPCR | | ++ | |
| MN054 tissue | n/a, R posterior sinus | | I | | F | | 58 | | qPCR | | ~ | |
| MN055 tissue | n/a, L frontal | | I | | F | | 50 | | qPCR | | ~ | |
| MN056 cells | n/a, R posterior fossa | | I | | F | | 61 | | qPCR | | ++ | |
| MN057 cells | Meningothelial, L parietal | | I | | M | | 58 | | qPCR | | +++ | |
| MN058 tissue | Angiomatous, R frontal | | I | | F | | 65 | | qPCR | | ~ | |
| MN062 cells | Meningothelial, olfactory groove | | I | | F | | 43 | | qPCR | | ++ | |
| MN066 cells | Psammomatous, thoracic | | I | | M | | 83 | | qPCR | | ++ | |
| MN071 cells | Secretory/angiomatous, R petroclivalppetrocicpetroclivalpetroclival | | I | | F | | 52 | | qPCR | | ++ | |
| MN073 cells | Fibroblastic, L convexity | | I | | F | | 70 | | qPCR | | ++ | |
| MN074 cells | Transitional, R angular gyrus | | I | | F | | 37 | | qPCR | | + | |
| MN075 tissue/cells | Transitional, R parietal | | I | | F | | 79 | | qPCR | | ~ | |
| MN076 tissue/cells | Atypical, olfactory groove | | II | | F | | 53 | | WB, qPCR | | +++ | |
| MN077 cells | Transitional, bilateral parasagittal | | I | | F | | 66 | | qPCR | | +++ | |
| MN078 cells | Transitional, L frontal | | I | | M | | 70 | | qPCR | | +++ | |
| MN079 tissue/cells | Atypical, occipital | | II | | M | | 75 | | qPCR | | ~ | |
| MN080 cells | Fibrous, L petrous | | I | | F | | 64 | | WB, qPCR | | +++ | |
| MN082 cells | Fibroblastic, R tentorial | | I | | F | | 57 | | qPCR | | +++ | |
| MN085 cells | Psammomatous/ fibrous, L frontal  L frontal | | I | | F | | 56 | | qPCR | | +++ | |
| MN087 tissue | n/a, CPA | | I | | M | | 47 | | qPCR | | ~ | |
| MN088 tissue | Transitional, sphenoid wing | | I | | F | | n/a | | qPCR | | ~ | |
| MN089 cells | Transitional, L parasagittal | | I | | M | | 53 | | qPCR | | +++ | |
| MN091 cells | n/a, L sphenoid wing | | I | | F | | 62 | | qPCR | | +++ | |
| MN092 cells | Microcystic, R convexity | | I | | F | | 59 | | qPCR | | +++ | |
| MN097 tissue | Atypical, L parasagittal recurrent | | II | | F | | 66 | | WB, qPCR | | +++ | |
| MN101 tissue | Atypical, R frontal | | II | | F | | 51 | | qPCR | | ++ | |
| MN102 cells | n/a, L frontal convexity | | I | | F | | 56 | | qPCR | | +++ | |
| MN104 tissue | Atypical, R paracentral | | II | | F | | 37 | | qPCR | | ~ | |
| MN105 tissue | Atypical, R frontal | | II | | F | | n/a | | qPCR | | ~ | |
| MN106 cells | Psammomatous, planum sphenoid sphnoidspheroidale | | I | | F | | 46 | | qPCR | | + | |
| MN107 cells | Transitional , R sphenoid wing | | I | | M | | 77 | | qPCR | | +++ | |
| MN109 cells | Transitional, L posterior frontal | | I | | F | | 48 | | qPCR | | +++ | |
| MN110 cells | Transitional, L lateral ventricle | | I | | F | | 47 | | qPCR | | ++ | |
| MN113 cells | Secretory, R temporal | | I | | F | | 52 | | qPCR | | +++ | |
| MN114 cells | Meningothelial, L parasagittal convexity | | I | | M | | 62 | | qPCR | | +++ | |
| MN115 tissue | Large cystic falcine | | I | | M | | 69 | | qPCR | | + | |
| MN125 tissue | Secretory, L petroclival | | I | | F | | 63 | | qPCR | | +++ | |
| MN133 tissue | Meningothelial, R fronto-parietal | | I | | M | | 87 | | WB | | ++ | |
| MN139 tissue | Transitional, R sphenoid wing | | I | | n/a | | n/a | | qPCR | | + | |
| MN140 tissue | n/a, Transitional | | I | | M | | 74 | | qPCR | | ++ | |
| MN148 tissue | Atypical, R frontal | | II | | M | | 79 | | qPCR | | ~ | |
| MN149 tissue | Meningothelial, R frontal | | I | | n/a | | n/a | | qPCR | | ~ | |
| MN157 tissue | Meningothelial,frontalfrontal | | I | | F | | n/a | | qPCR | | ~ | |
| MN168 tissue | Atypica, R occipital | | II | | n/a | | n/a | | qPCR | | + | |
| MN170 tissue | Meningothelial, frontal parafalcine | | I | | F | | 70 | | WB, qPCR | | + | |
| MN176 tissue | Microcystic, L frontal convexity | | I | | F | | 43 | | WB | | ~ | |
| MN180 tissue | Transitional, R occipital lobe | | I | | F | | 45 | | WB, qPCR | | ++ | |
| MN182 tissue | Atypical, R fronto-parietal | | II | | F | | 66 | | qPCR | | ~ | |
| MN183 tissue | Chordoid, sellar region | | II | | F | | 75 | | qPCR | | ~ | |
| MN186 tissue | Anaplastic, R temporal | | III | | M | | 62 | | qPCR | | ~ | |
| MN188 tissue | Fibrous, poster fossa | | I | | F | | 33 | | qPCR | | ~ | |
| MN189 tissue | Atypical, left lateral ventricle | | II | | M | | 55 | | qPCR | | ++ | |
| MN194 tissue | Atypical, occipital | | II | | F | | 41 | | qPCR | | ~ | |
| MN196 tissue | Atypical, L parafalcine | | II | | M | | 39 | | qPCR | | ~ | |
| MN200 tissue | Atypical, L fronto-parietal | | II | | n/a | | 66 | | qPCR | | +++ | |
| MN208 tissue | Psammomatous, thoracic vspinespie | | I | | F | | n/a | | qPCR | | ~ | |
| MN214 tissue | Meningothelial, olfactory groove | | I | | F | | n/a | | qPCR | | ~ | |
| MN217 tissue | Fibrous, R tentorial | | I | | n/a | | n/a | | qPCR | | ++ | |
| MN219 tissue | Atypical, L fronto-parafalcine | | II | | M | | 55 | | qPCR | | ~ | |
| MN225 tissue | Atypical, L fronto-parafalcine | | II | | M | | 57 | | qPCR | | ~ | |
| MN234 tissue | Atypical, R fronto-parietal | | II | | F | | 79 | | qPCR | | + | |
| MN235 tissue | Atypical, R fronto-parafalcine | | II | | F | | 71 | | qPCR | | ~ | |
| MN242 tissue | Fibrous, olfactory groove | | I | | F | | n/a | | qPCR | | ~ | |
| MN248 tissue | Transitional, frontal | | I | | M | | n/a | | qPCR | | + | |
| MN251 tissue | Fibrous, tentorial | | I | | F | | n/a | | qPCR | | ~ | |
| MN252 tissue | Atypical, R parasagittal | | II | | F | | 73 | | qPCR | | ~ | |
| MN261 tissue | Transitional, L parasagittal | | I | | M | | n/a | | qPCR | | + | |
| MN263 tissue | Atypical, L frontal convexity | | II | | M | | 78 | | qPCR | | ~ | |
| MN274 tissue | Fibrous, L parietal | | I | | F | | 68 | | qPCR | | +++ | |
| MN278 tissue | Meningothelial, R sphenoid sssphemnoid | | I | | F | | n/a | | qPCR | | + | |
| MN332 tissue | L frontal parafalcine | | II | | M | | 68 | | qPCR | | ~ | |
| MN338 tissue | L temporal convexity | | II | | F | | 88 | | qPCR | | ++ | |
| NH09 tissue | Anaplastic, occipital | | III | | n/a | | n/a | | qPCR | | +++ | |
| NH10 tissue | Anaplastic, frontal | | III | | n/a | | n/a | | qPCR | | +++ | |
| J1 tissue | Atypical, sphenoid wing | | II | | F | | 62 | | IHC | | ++ | |
| J2 tissue | Atypical, parafalcine | | II | | F | | 51 | | WB, IHC | | ++ | |
| J3 tissue | Atypical, frontal | | II | | M | | 64 | | WB, IHC | | ++ | |
| J4 tissue | Atypical brain invasion, occipital | | II | | M | | 66 | | WB, IHC, qPCR | | +++ | |
| J5 tissue | Fibroblastic, occipital | | I | | F | | 50 | | WB, IHC | | +++ | |
| J6 tissue | Transitional, parasagittal | | I | | F | | 37 | | WB, IHC | | +++ | |
| J7 tissue | Transitional, parasagittal | | I | | F | | 72 | | WB, IHC | | +++ | |
| J8 tissue | Transitional, parasagittal | | I | | F | | 68 | | WB, IHC | | ++ | |
| J9 tissue | Malignant, occipital | | III | | F | | 82 | | WB, IHC | | ++ | |
| J10 tissue | Malignant occipital | | III | | M | | 85 | | WB, IHC, qPCR | | ++ | |
| J11 tissue | Malignant, occipital | | III | | M | | 85 | | WB, IHC, qPCR | | +++ | |
| J12 tissue | Malignant, parasagittal | | III | | M | | 87 | | WB, IHC | | ++ | |
| J22 tissue | Atypical, occipital | | II | | M | | 69 | | qPCR | | ++ | |
| J23 tissue | Atypical, temporal | | II | | F | | 62 | | qPCR | | ++ | |
| D1 tissue | Meningothelial | | I | | n/a | | n/a | | IHC | | + | |
| D2 tissue | Meningothelial | | I | | n/a | | n/a | | IHC | | + | |
| D3 tissue | Meningothelial | | I | | n/a | | n/a | | IHC | | + | |
| D4 tissue | Secretory | | I | | n/a | | n/a | | IHC | | +++ | |
| D5 tissue | Secretory | | I | | n/a | | n/a | | IHC | | +++ | |
| D6 tissue | Secretory | | I | | n/a | | n/a | | IHC | | ++ | |
| D7 tissue | Secretory | | I | | n/a | | n/a | | IHC | | +++ | |
| D8 tissue | Secretory | | I | | n/a | | n/a | | IHC | | +++ | |
| D9 tissue | Secretory | | I | | n/a | | n/a | | IHC | | + | |
| D10 tissue | Transitional | | I | | n/a | | n/a | | IHC | | ++ | |
| D11 tissue | Mosaic Fibroblastic | | I | | n/a | | n/a | | IHC | | + | |
| D12 tissue | Fibroblastic | | I | | n/a | | n/a | | IHC | | ++ | |
| D13 tissue | Fibroblastic | | I | | n/a | | n/a | | IHC | | ++ | |
| D14 tissue | Psammomatous, spinal | | I | | n/a | | n/a | | IHC | | ++ | |
| D15 tissue | Atypical | | II | | n/a | | n/a | | IHC | | ++ | |
| D16 tissue | Atypical | | II | | n/a | | n/a | | IHC | | ++ | |
| D17 tissue | Atypical, brain invasion | | II | | n/a | | n/a | | IHC | | ++ | |
| D18 tissue | Atypical, brain invasion | | II | | n/a | | n/a | | IHC | | ++ | |
| D19 tissue | Atypical, brain invasion | | II | | n/a | | n/a | | IHC | | ++ | |
| D20 tissue | Malignant | | III | | n/a | | n/a | | IHC | | ++ | |
| D21 tissue | Malignant | | III | | n/a | | n/a | | IHC | | + | |
| D22 tissue | Malignant | | III | | n/a | | n/a | | IHC | | + | |
| D23 tissue | Malignant | | III | | n/a | | n/a | | IHC | | +++ | |
| D24 tissue | Malignant | | III | | n/a | | n/a | | IHC | | ++ | |
| D25 tissue | Malignant | | III | | n/a | | n/a | | IHC | | +++ | |
| HMC | Human meningeal cells | | n/a | | n/a | | n/a | | WB, qPCR, IF | | Control | |
| BioChain^R1234043-10^ | Cerebral meninges | | n/a | | F | | 82 | | qPCR | | Control | |
| ABS^150102416^ | Cerebral meninges | | n/a | | F | | 92 | | WB, qPCR | | Control | |
| ABS^60200003215^ | Cerebral meninges | | n/a | | F | | 78 | | WB, qPCR | | Control | |
| C1 | Cerebral meninges | | n/a | | n/a | | n/a | | IHC | | Control | |
| C2 | Cerebral meninges | | n/a | | n/a | | n/a | | IHC | | Control | |
| C3 | Cerebral meninges | | n/a | | n/a | | n/a | | IHC | | Control | |
| n/C4 | Cerebral meninges- glioma | | n/a | | n/a | | n/a | | IHC | | Control | |
| n/C5 | Cerebral meninges- glioma | | n/a | | n/a | | n/a | | IHC | | Control | |
| n/C6 | Cerebral meninges- glioma | | n/a | | n/a | | n/a | | IHC | | Control | |
| Abcam^ab29466^ | Brain (human) tissue lysate | | n/a | | n/a | | n/a | | WB | | + | |
| n/C7 | Normal brain temporal lobe | | n/a | | n/a | | n/a | | IHC | | Control | |
| n/C8 | Normal brain temporal lobe | | n/a | | n/a | | n/a | | IHC | | + | |
| n/C9 | Normal brain occipital lobe | | n/a | | n/a | | n/a | | IHC | | Control | |
| n/C10 | Normal brain frontal lobe | | n/a | | n/a | | n/a | | IHC | | Control | |
