# Supplementary material for: Constitutive activation of the EGFR–STAT1 axis increases proliferation of meningioma tumor cells
Source: Neurooncol Adv. 2020 Jan 21;2(1):vdaa008. doi: 10.1093/noajnl/vdaa008 (PMC7212880; doi:10.1093/noajnl/vdaa008)
Supplement: vdaa008_suppl_Supplementary_Table_S2 [file vdaa008_suppl_supplementary_table_s2.docx]

**Supplementary Table 2.** Complete list of the antibodies employed in the study, their application and the concentrations used. WB: Western Blot; IF: Immunofluorescence; IP: Immunoprecipitation; IHC: Immunohistochemistry.

| **Antibody** | **Manufacturer** | **Application** | **Dilution** |
| --- | --- | --- | --- |
| STAT1 | Cell Signaling Technology - #9172  Santa Cruz Biotechnology - sc-592 | WB  WB  IF  IHC | 1:1000  1:1000  1:300  1:150 |
| pSTAT1-Y701 | Abcam - ab29045  R&D Systems - AF2894  Cell signalling - #7649 | WB  IF  WB  IHC  WB  IP | 1:500  1:100  1:1000  1:200  1:500  1:50 |
| pSTAT1-S727 | Cell Signaling Technology - #9177 | WB  IF  IHC | 1:1000  1:100  1:400 |
| JAK1 | Cell Signaling Technology - #3344 | WB | 1:1000 |
| pJAK1- Y1022/1023 | Cell Signaling Technology - #3331 | WB | 1:500 |
| JAK2 | Cell Signaling Technology - #3230 | WB | 1:1000 |
| pJAK2- Y1007/1008 | Cell Signaling Technology - #3771 | WB | 1:500 |
| TYK2 | Cell Signaling Technology - #14193 | WB | 1:500 |
| pTYK2- Y1054/1055 | Cell Signaling Technology - #9321 | WB | 1:500 |
| IFN γ | Abcam - ab25101 | WB | 1:500 |
| CD163 | Bio-Rad - MCA1853 | WB | 1:500 |
| Merlin | Cell Signaling Technology - #6995 | WB | 1:1000 |
| pMerlin- S518 | Cell Signaling Technology - #9163 | WB | 1:500 |
| ERK | Cell Signaling Technology - #4695 | WB | 1:2000 |
| pERK- T202/204 | BD Biosciences - #612358 | WB | 1:500 |
| AKT1 | Cell Signaling Technology - #4691 | WB | 1:1000 |
| pAKT1- S473 | Cell Signaling Technology - #9271 | WB | 1:500 |
| RB | Cell Signaling Technology - #9309 | WB | 1:2000 |
| pRB- S780 | Cell Signaling Technology - #8180 | WB | 1:1000 |
| CD63 (MEM-259) | Thermo Fisher Scientific - MA119281 | IF | 1:250 |
| CD63 | Cambridge Bioscience - EXOAB-CD63A-1 | WB | 1:500 |
| CD9 (C-4) | Santa Cruz Biotechnology - #13118 | IF | 1:250 |
| CD9 | Cell Signaling Technology - #13174 | WB | 1:500 |
| GM130 | BD Transduction Laboratories - #610823 | WB | 1:1000 |
| Calnexin (H-70) | SantaCruz Biotechnology - #11397 | WB | 1:1000 |
| CyclinD1 | Cell Signaling Technology - #2978 | WB | 1: 300 |
| Ki67 (MIB-1) | DAKO - #M7240 | IF | 1: 1000 |
| PIAS1 | Cell Signaling Technology - #3550 | WB | 1:1000 |
| PIAS3 | Cell Signaling Technology - #9042 | WB | 1:1000 |
| PIAS4 | Cell Signaling Technology - #4392 | WB | 1:1000 |
| SOCS1 | Cell Signaling Technology - #3950 | WB | 1:1000 |
| SOCS2 | Cell Signaling Technology - #2779 | WB | 1:1000 |
| SOCS3 | Cell Signaling Technology - #2932 | WB | 1:1000 |
| EGFR | Cell Signaling Technology - #4267 | WB | 1:1000 |
| pEGFR- Y1068 | Cell Signaling Technology - #3777 | WB | 1:500 |
| pP70 S6K – T421/S424 | Cell Signaling Technology - #9204 | WB | 1:500 |
| P70 S6K | Cell Signaling Technology - #9202 | WB | 1:500 |
| GAPDH | EMD Millipore – MAB374 | WB | 1:50000 |
