# Supplementary material for: Constitutive activation of the EGFR–STAT1 axis increases proliferation of meningioma tumor cells
Source: Neurooncol Adv. 2020 Jan 21;2(1):vdaa008. doi: 10.1093/noajnl/vdaa008 (PMC7212880; doi:10.1093/noajnl/vdaa008)
Supplement: vdaa008_suppl_Supplementary_Table_and_Figure_Legend [file vdaa008_suppl_supplementary_table_and_figure_legend.docx]

**Supplementary Figure Legends**

**Supplementary Fig. 1** Purity of primary meningioma cultures and genetic background of our cohort of samples. **A** Representative flow cytometry analysis of primary MN cells. Dot plots show 10,000 live cells and represent monocyte marker, CD14-PerCP-Cy5.5 (FL3 channel) and potential meningioma tumour marker, CD44-APC (FL4 channel). Upper right quadrant represents CD14+ CD44+ phenotype and decreases with passage number (CD14+ CD44+ % = Passage 0-4.6, Passage 1-2.1, Passage 2-0.1, Passage 3-0.2). Data analysed was performed on Flow Jo version10.0. **B** Representative confocal images of three primary MN cells tested at passage 3, homogeneously positive for the meningioma marker vimentin (green), while negative for the fibroblast marker CD90. Scale bar 10 μm. Nuclei were stain with DAPI (blue). **C** WB analysis showing the expression of Merlin in different grade meningiomas *vs.* NMT; Next Generation Sequencing (NGS) confirmed that only samples J8, J1 and J2 were Merlin-positive not having any mutation on *Merlin* or loss of heterozygosity (LOH). **D** WB showing the expression of Merlin in in BM-1 and tumour-derived MN cells *vs.* HMC.

**Supplementary Fig. 2** The JAK/STAT pathway in meningioma cells can be activated by IFNα. **A** WB analysis of STAT1 and pSTAT1 (Y701 and S727) protein levels in HMC and two primary MN cells, after IFNα treatment at the concentration of 50 ng/ml for the indicated amount of time. Phosho-JAK1 and pTYK2 are shown to confirm the activation of the JAK/STAT pathway. **B** Representative confocal z-stack images showing localization of pSTAT1-Y701 (green) and pSTAT1-S727 (red) in primary MN cells before and after IFNα stimulation (50 ng/ml for 1 h). Scale bar 50 μm. Nuclei were stain with DAPI (blue). **C** Representative WB conducted in primary MN cells showing higher levels of JAK1, when compared to HMC.

**Supplementary Fig. 3** STAT1 knocked-down reduces proliferation of BM-1 meningioma cells. **A** WB analysis showing the reduction in STAT1 protein levels after *STAT1* sh-RNA-mediated silencing compared to scramble control. **B** Reduction in *STAT1* gene expression associated to *STAT1* sh-RNA-mediated silencing compared to scramble control. Data are presented as mean ± SEM; * = p≤ 0.05. **C** Representative images of the immunofluorescent staining of STAT1 (green) and the proliferation marker Ki67 (red) after *STAT1* sh-RNA-mediated silencing compared to scramble control. Nuclei are stain with DAPI (blue). **D** Histogram presenting the statistical reduction of proliferating cells after STAT1-KD compared to scramble control. Data are presented as mean ± SD; * = p≤ 0.05.

**Supplementary Fig. 4** WB quantification after canertinib treatment in primary MN cells. Detailed WB quantification for the histograms presented in Fig 5E. Protein expression was quantified after normalising for the corresponding GAPDH amount and is presented as fold change of the vehicle-treated sample (V).
